# The law of surprise: temporal variation and seasonality of acute aortic syndromes: a single-centre epidemiologic cohort analysis spanning 20 years

**DOI:** 10.1186/s13019-026-04750-1

**Published:** 2026-11-03

**Authors:** Raphael Groß, Mohamed Elbayomi, Frank Harig, Friedrich Mellert, Robert Cesnjevar, Presheet Pathare

**Affiliations:** Department of Cardiac Surgery, Friedrich-Alexander-University, Krankenhausstr. 12, 91054 Erlangen, Bavaria Germany

**Keywords:** Aortic dissection, Ascending aorta, Risk stratification, Seasonality, Epidemiologic study

## Abstract

**Background and aims:**

Acute aortic syndromes (AAS) represent life-threatening cardiovascular emergencies characterized by high mortality rates despite rapid intervention. Global epidemiological data indicate substantial variance in incidence. Longitudinal single-centre data over extended periods are limited but crucial for understanding diagnostic, demographic, and systemic factors influencing admission trends.(1). This study aimed to analyze annual and seasonal variation trends of AAS over a 20-year period in a single maximal care centre and explore potential chronological and environmental contributors to this variation.

**Methods:**

This retrospective cohort study evaluated 403 patients admitted with a confirmed diagnosis of AAS between 2005 and 2025 at the University Hospital Erlangen, Germany. All diagnoses were established via computed tomography angiography (CTA) and confirmed intraoperatively. Annual, quarterly, and monthly surgical volumes were calculated, and temporal trends were analyzed using Poisson and negative binomial regression models. Admission rates during public holidays and school vacations were also evaluated.

**Results:**

Over the 20-year observation period, a steady increasing trend in the annual number of AAS cases was observed, peaking in 2017. Analysis of quarterly distributions revealed a statistically significant dominance of cases in the fourth quarter (Q4: October–December; *p* < 0.05) compared to the second (Q2) and third (Q3) quarters. Monthly analysis demonstrated a prominent peak in October (incidence rate ratio [IRR] = 1.85 vs. June, *p* < 0.05). Conversely, fixed public holidays and school vacations were associated with statistically significant reductions in daily admission counts (*p* < 0.05), with the lowest relative case counts observed during the Christmas vacation period (IRR: 0.40–0.50).

**Conclusion:**

The case counts of acute aortic syndromes exhibits significant temporal and seasonal variation, characterized by a distinct late-autumn and early-winter peak, alongside significant declines during institutional vacation periods. These findings suggest that acute cardiovascular events are heavily modulated by cyclical environmental and socio-systemic factors.

## Introduction

### Background

Despite notable advancements in preventing, diagnosing, and managing acute aortic syndromes, it persists as a challenging cardiovascular condition marked by elevated early mortality rates and significant ongoing health complications for those who endure the initial episode [1].

Numerous international registries have highlighted seasonal variations in the presentation of acute cardiovascular events, frequently noting an increased admission densities during colder months. However, single-centre longitudinal data spanning multiple decades remain sparse. Granular, localized cohort analyses are highly valuable for identifying subtle temporal patterns, demographic shifts, and healthcare infrastructure impacts that widespread registry datasets may smooth out [2].

The primary objective of this study was to conduct a retrospective cohort analysis of the annual and seasonal admission densities of AAS requiring operative therapy at a single maximal care tertiary referral center (University Hospital Erlangen, Germany) over a 20-year period (2005–2025). Furthermore, we sought to scrutinize the physiological, institutional, and environmental drivers underlying these temporal fluctuations, providing a comprehensive assessment of seasonal peaks and holiday-related declines to optimize institutional resource allocation and preventive vigilance [3]. The assumption is that, acute aortic syndromes should occur randomly, but seldom do.

### Objectives

To conduct a retrospective cohort analysis the variation of admissions of acute aortic syndromes (specifically, but not limited to Standford Type A Aortic dissections, “non-A/non-B” aortic dissections and the respective intramural hematomas) requiring operative therapy in a single centre in a maximal care setting (University Hospital Erlangen, Germany). The focus of the study was to examine the annual case counts, along with examination of probable causes and the possible local and societal factors that may affect this variation.

## Methods

### Study design and patient population

#### Aims and study design

The primary objective was to assess the admission trends to see if there was a statistically significant variation in the annual distribution of patient admissions for aortic dissections. The secondary objectives were to analyse the trends to identify a correlation with respect to local holidays and identify annual trends with respect to various subdivisions.

#### Setting

Patients with a confirmed diagnosis of an acute aortic syndrome who received operative therapy between April 2005 and November 2025 (year of analysis) were included. All patients received an initial diagnosis through CT imaging. The diagnosis was confirmed intra-operatively.

#### Participant patient population

Inclusion criteria: Inclusion Criteria: Adult patients (> 18 years) with a verified preoperative diagnosis of an Acute Aortic Syndrome including: Stanford Type A aortic dissection, “non-A/non-B” aortic dissection (dissections with the primary intimal tear located within Ishimaru zones 1–2), acute rupture of the ascending aorta or aortic arch, or acute intramural hematoma (IMH) displaying high-risk features (e.g., penetrating aortic ulcers) requiring emergency surgical reconstruction. All diagnoses were established via contrast-enhanced Computed Tomography Angiography (CTA) and definitively confirmed intraoperatively.

Exclusion criteria: Patients with a history of prior cardiac surgery or median sternotomy, chronic aortic syndromes, retrograde extensions of aortic dissection secondary to prior interventions for Type B dissections, cases where intraoperative findings disproved the primary diagnosis, and patients with missing or incomplete clinical data.

#### Data sources

 Internal hospital electronic health records and immediate postoperative admission databases were utilized to extract patient baseline demographic characteristics (age, biological sex, height, and weight) and the exact timestamp of emergency admission. Regional socioeconomic and academic calendars—encompassing specific dates for Bavarian public holidays, school vacations (Christmas, Winter/Spring, Easter, Pentecost, Summer, and Autumn), and weekends—were obtained from official repositories of the Bavarian State Ministry for Education and Cultural Affairs (Bayerisches Staatsministerium für Unterricht und Kultus) and verified against schulferien.org.

#### Statistical methods

Descriptive statistics were calculated for all baseline patient characteristics; continuous variables are presented as means with ranges, and categorical variables are reported as absolute numbers and percentages. Long-term temporal variations in case numbers were evaluated using standard deviations, 95% confidence intervals (CI), and trendline modeling.

To evaluate the seasonal and cyclical distribution of admissions, quarterly total counts were compared using a One-Way Analysis of Variance (ANOVA) and the non-parametric Kruskal–Wallis test, followed by Tukey’s Honest Significant Difference (HSD) post-hoc test for pairwise comparisons. To model monthly admission counts and account for potential overdispersion, case distributions were evaluated using Poisson regression and Negative Binomial regression models. June was predefined as the absolute reference baseline because it exhibited the lowest baseline case count. Incidence Risk Ratios (IRRs) and 95% CIs were calculated, and statistical significance was evaluated using Wald tests.

Daily admission densities during standard working days, public holidays, and specific vacation blocks were compared using a two-sided Welch’s t-test (accounting for unequal variances) and the non-parametric Mann–Whitney U test. Multiple testing comparisons were adjusted using the Bonferroni correction. Cohen’s d coefficients were calculated to assess the standardized effect sizes of holiday-related drops in admission density. All statistical tests were two-sided, and a value of *p* < 0.05 was considered statistically significant. Statistical computations were performed using Stata/SE statistical software (versions 17.0 and 19.0, StataCorp LLC, College Station, TX, USA).

### Human participant protection

This retrospective study complied with the Helsinki Declaration (2000), and the local Ethics Committee (EC, No.22-448-Br) approved this analysis based on retrospective data retrieval; the EC waived the need for written patients’ informed consent.

## Results

A total of 475 patients presenting with suspected AAS were initially assessed. Following the exclusion of patients with a history of prior cardiac surgery or sternotomy, chronic aortic syndromes, retrograde aortic dissection secondary to Type B treatment, non-confirmed intraoperative findings, or incomplete datasets, a final cohort of 403 patients was included in the analysis.

**Fig. 1:**
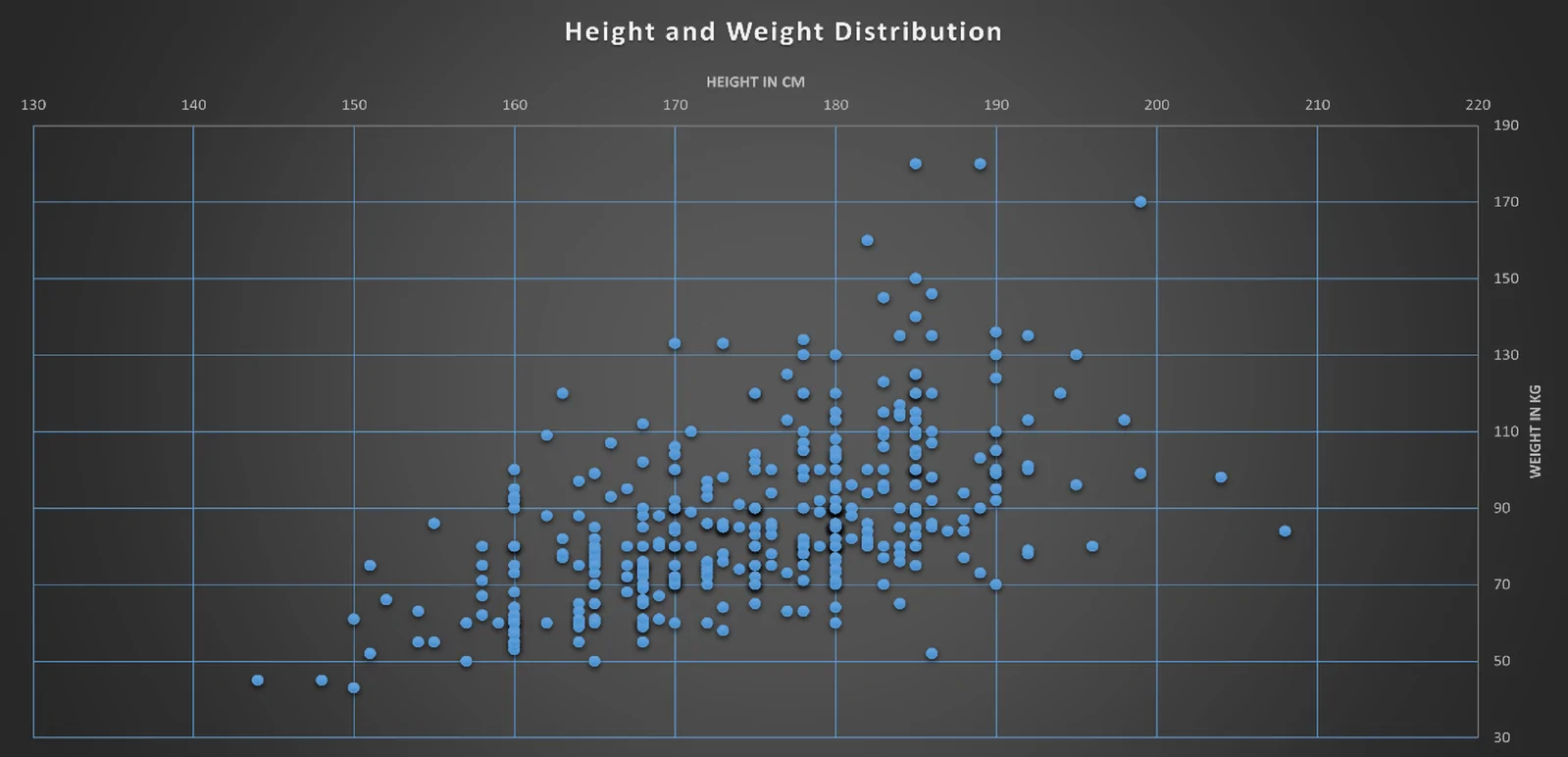
Scatter plot of the baseline patient characteristics with x=Height in cm, y=weight in Kg. Note the shift in 0 due to scaling and skewed distribution of data

The baseline demographic characteristics of the study cohort are presented in Table 1. The cohort exhibited a high prevalence of biological males (*n* = 275, 68.2%) compared to biological females (*n* = 128, 31.8%). The mean age at presentation was 58 years (range: 20–89 years). Anthropometric analysis revealed a mean height of 174.4 cm (range: 144–208 cm) and a mean weight of 87 kg (range: 43–180 kg), with a distinct cluster within average ranges and only 30 patients exceeding a height of 190 cm (Fig. 1).

**Table 1:** Baseline patient characteristics

Mean baseline patient characteristics (Range)	
Age in years	58 (20–89)
Female sex ⎯ no. (%)	128 (31.68)
Height in cm	174.4 (144–208)
Weight in kg	87 (43–180)

### Long-term annual and quarterly distribution trends

**Fig. 2:**
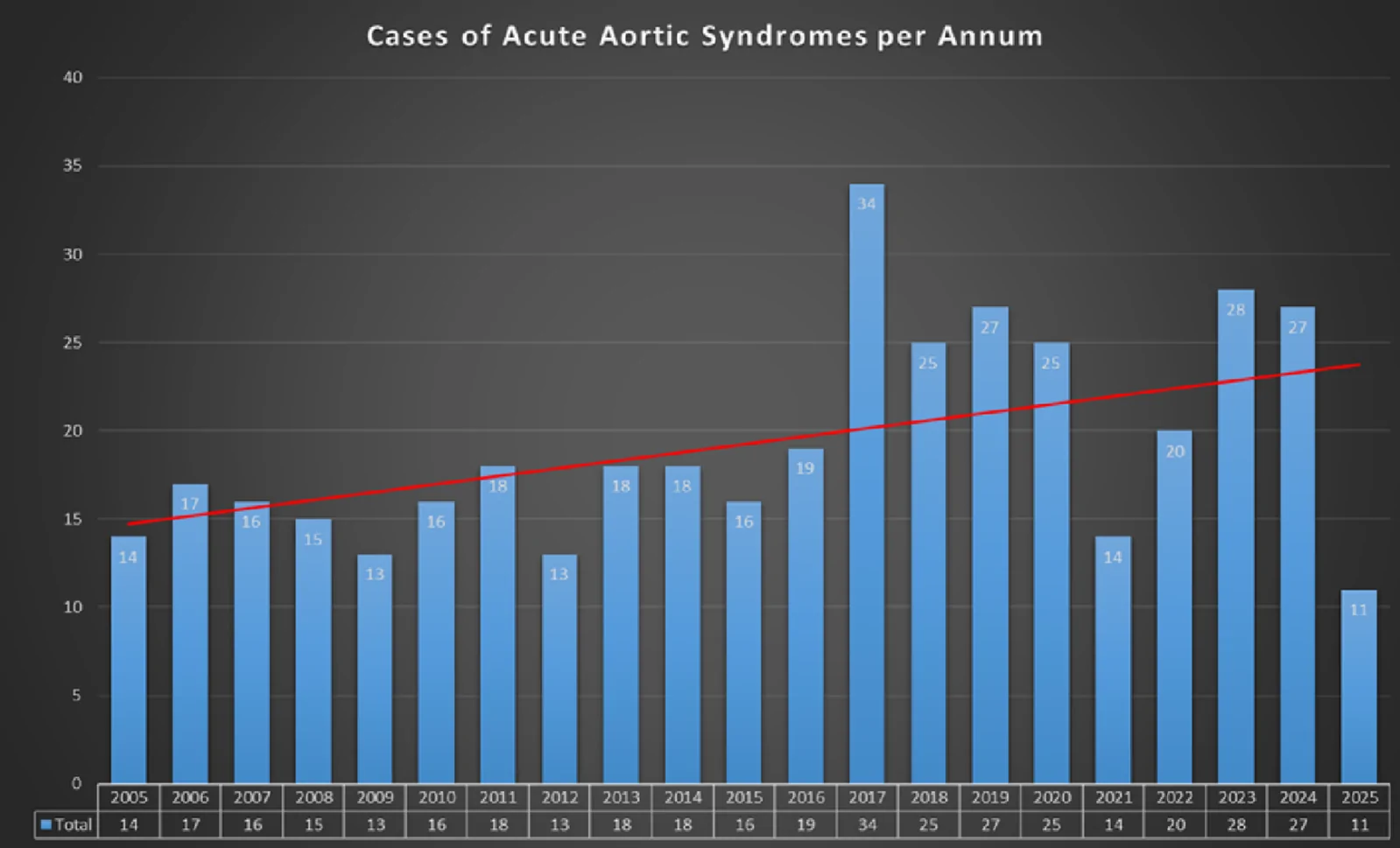
Annual distribution of cases of AAS. Note 2025 and 2005 are partially displayed due to observational and temporal restrictions in data analysis

Stratification of admissions by calendar year from 2005 to 2025 revealed a progressive, long-term upward trend in the absolute number of managed AAS cases (Fig. 2). The maximum annual volume was recorded in the year 2017 with 34 admissions, which represents a statistical outlier in the temporal series.

**Fig. 3:**
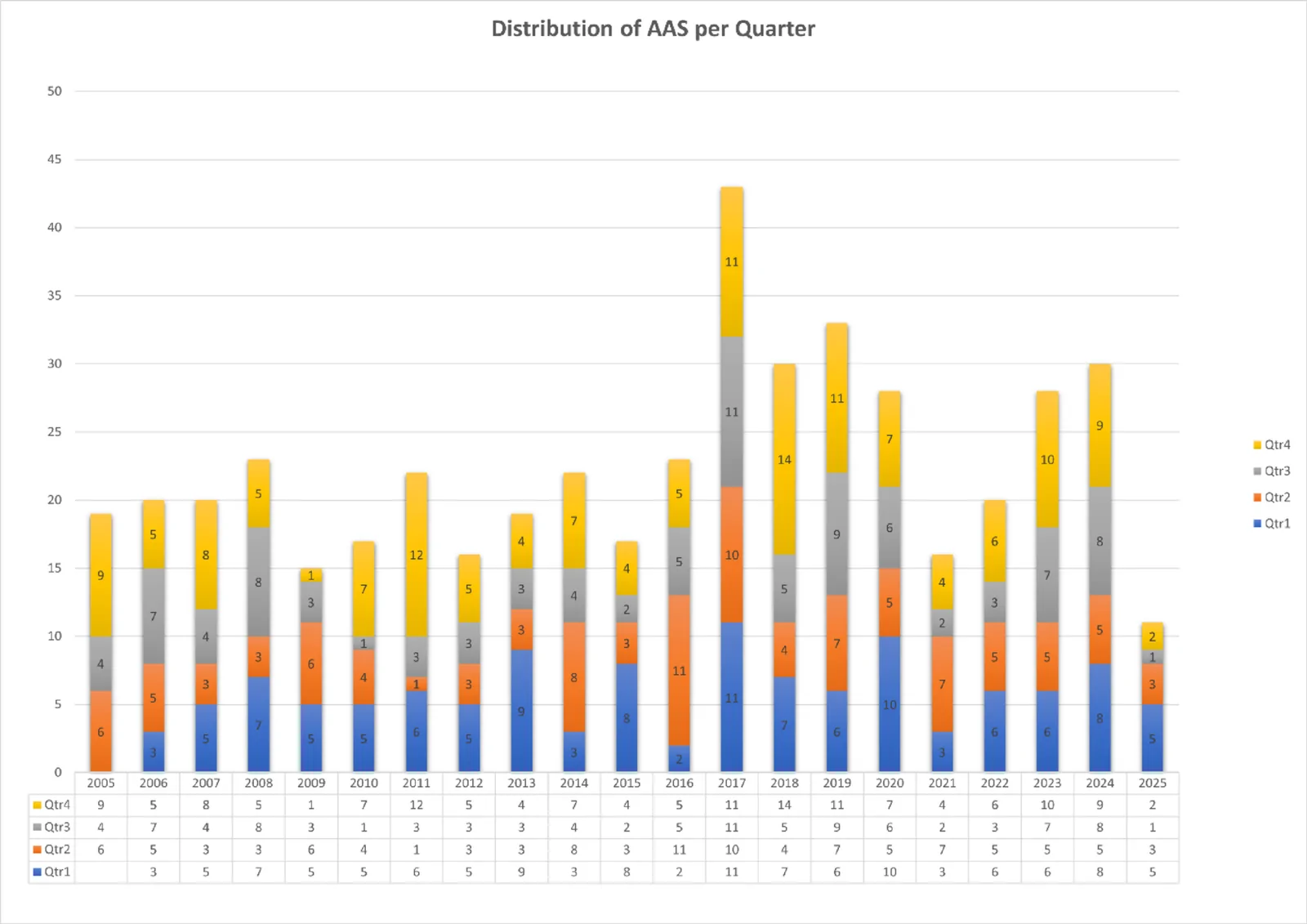
Distribution of AAS per quarter per year. Scale provided in the figure

To analyze cyclical variations within years, admissions were aggregated by fiscal quarters. One-Way ANOVA and Kruskal–Wallis tests demonstrated a highly non-uniform distribution across the seasons (*p* = 0.004). Pairwise comparisons using the Tukey HSD post-hoc test confirmed a significant dominance of cases during the fourth quarter (Q4: October to December), accumulating 131 total cases, representing a 30% increase over the expected uniform baseline distribution (mean per year = 6.24 ± 2.88, 95% CI: 5.01–7.47; *p* < 0.05) (Fig. 3). Quarters 1 through 3 exhibited lower surgical volume, with the absolute nadir observed in Q2 and Q3, while Q1 admissions were moderately elevated compared to the spring and summer periods.

### Monthly incidence risk ratios (IRR)

To achieve higher temporal granularity, a Poisson regression model and an overdispersion-adjusted negative binomial regression model were fitted to monthly case counts, utilizing June (the month with the lowest case volume, temporal annual median) as the reference point baseline. Calculated Incidence Risk Ratios (IRRs) represent relative ratios of case presentation density compared to the baseline month/quarter, rather than absolute population risk shifts. (Table 3).

**Table 3:** Monthly distribution of AAS and the relative case presentation density with June with the lowest number of cases as the reference

Months	Number of aortic dissections	IRR vs. June	% change vs. June	95% CI	*p*-value (Wald test)
Jan	39	1.5	50%	0.88–2.57	0.13
Feb	33	1.27	27%	0.74–2.19	0.38
Mar	26	1	0%	—	—
Apr	27	1.04	4%	0.59–1.82	0.89
May	35	1.35	35%	0.79–2.31	0.28
Jun	26	1.00 (ref)	—	—	—
Jul	30	1.15	15%	0.67–1.99	0.63
Aug	31	1.19	19%	0.70–2.04	0.52
Sep	26	1	0%	—	—
Oct	47	1.85	85%	1.08–3.18	0.024
Nov	42	1.62	62%	0.95–2.78	0.075
Dec	41	1.58	58%	0.92–2.72	0.094

**Fig. 4:**
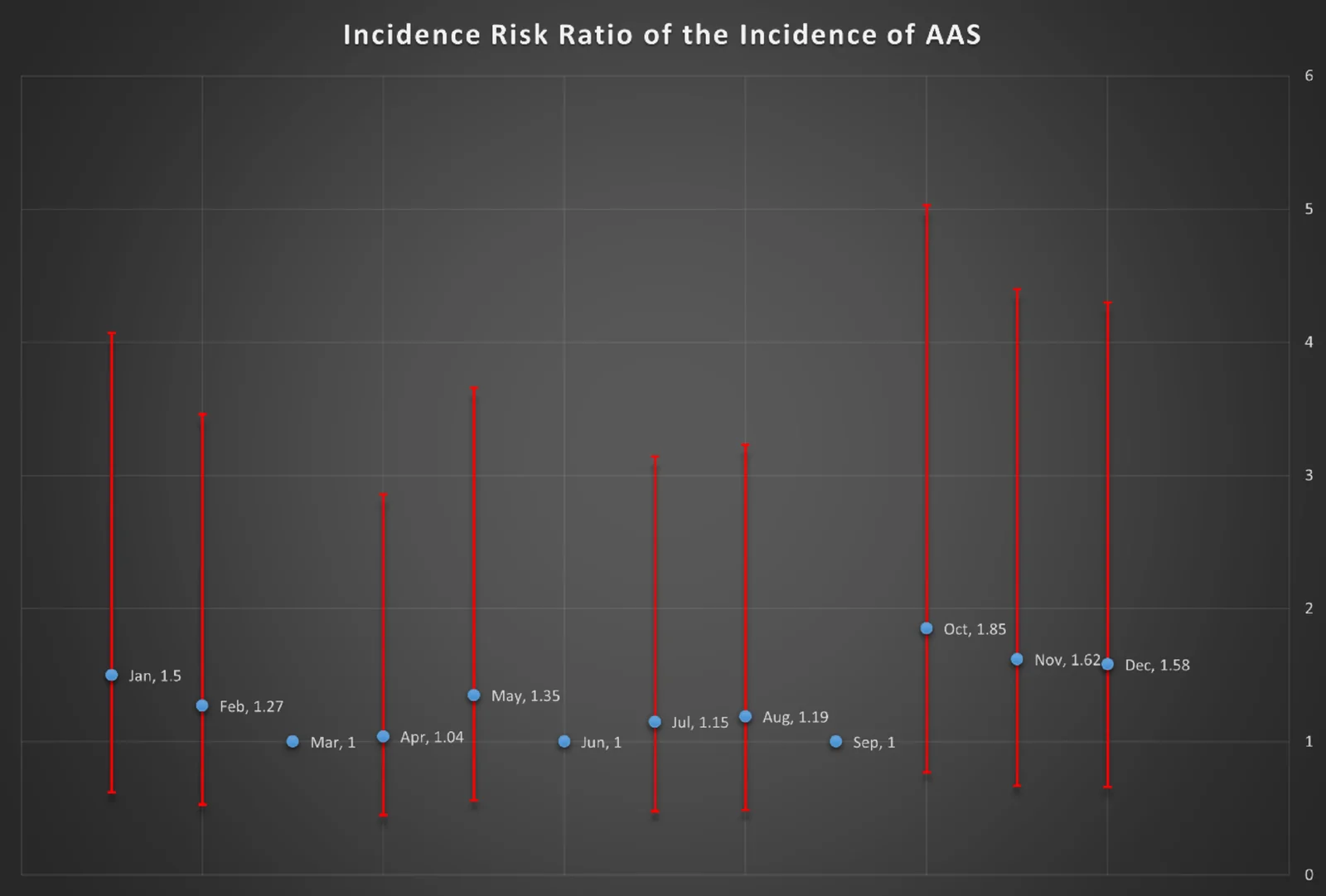
Incidence risk ratio of AAS per month with June having the lowest admissions as the reference

A pronounced surge in admissions was observed in late autumn and winter. The admissions of AAS peaked significantly in October, showing an 85% increase compared to June (IRR = 1.85, 95% CI: 1.08–3.18, *p* = 0.024) (Fig. 4). This peak was sustained into November (IRR = 1.62, 95% CI: 0.95–2.78, *p* = 0.075) and December (IRR = 1.58, 95% CI: 0.92–2.72, *p* = 0.094), both demonstrating borderline statistical significance. A secondary minor increase was observed in January (IRR = 1.50, 95% CI: 0.88–2.57, *p* = 0.13), whereas March and September aligned exactly with the June reference baseline.

**Fig. 5:**
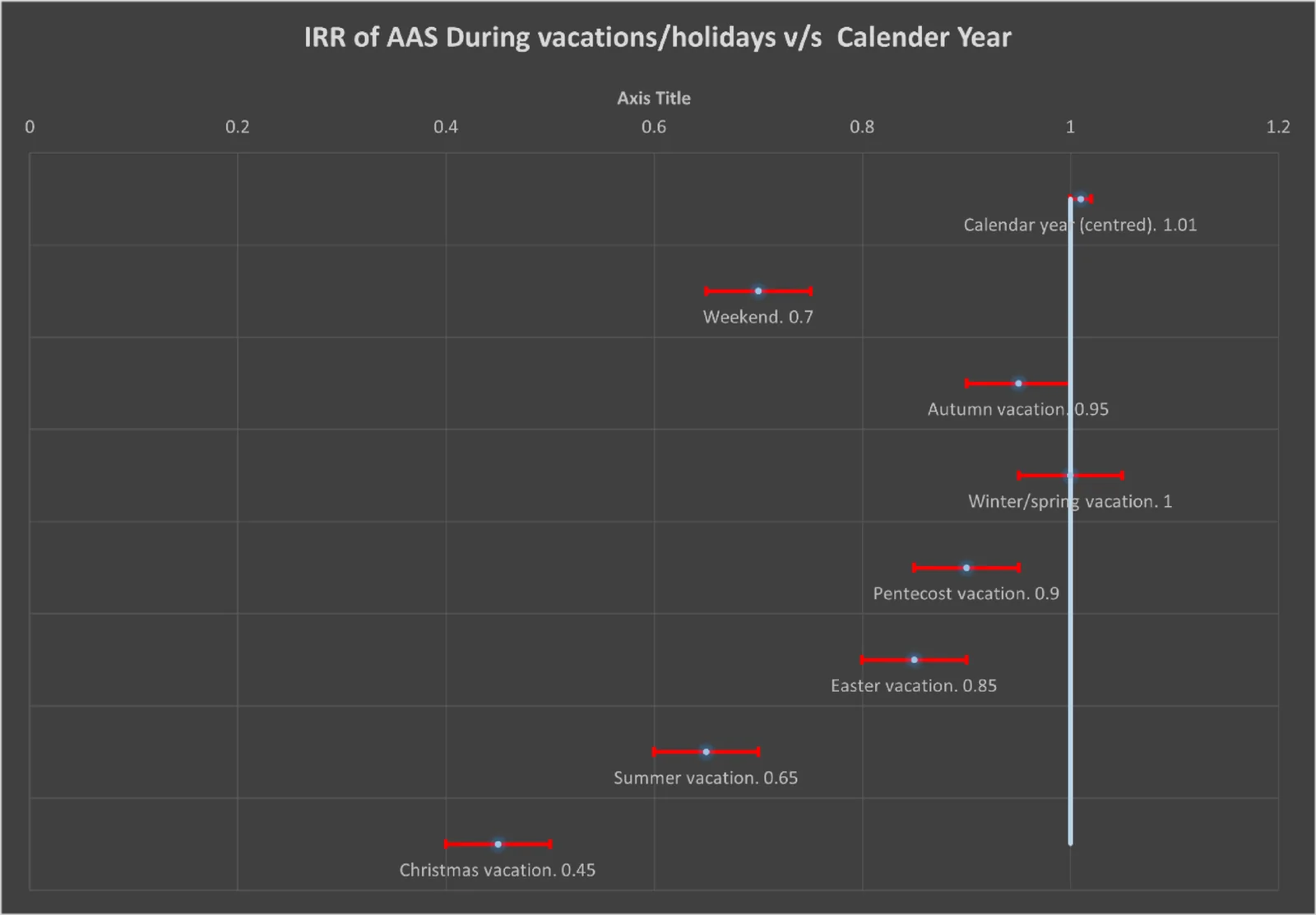
Relative cases of AAS vacation/weekends compared to calendar year as reference

As summarized in Table 2 and illustrated in Fig. 5, the most severe declines occurred during the Christmas vacation period, with an admission IRR of 0.40–0.50 (*p* < 0.001), followed by the summer vacation block (IRR ≈ 0.60–0.65, *p* < 0.01). Weekend presentations also demonstrated a noTable 30% reduction compared to regular weekdays (IRR ≈ 0.70, *p* < 0.001).

**Table 2:** Relative case presentation density of AAS vacation/weekends compared to calendar year as reference

Variable	IRR (poisson)	IRR (negative binomial)	*p* value
Christmas vacation	0·40–0·50	~ 0·45	< 0·001
Summer vacation	~ 0·60	~ 0·65	< 0·01
Easter vacation	0·80–0·90	~ 0·85	< 0·05
Pentecost vacation	0·85–0·95	~ 0·90	0·05–0·10
Winter / spring vacation	~ 1·00	~ 1·00	0·05–0·10
Autumn vacation	~ 0·95	~ 0·95	0·05–0·10
Weekend (vs. weekday)	~ 0·70	~ 0·70	< 0·001
Calendar year (centred)	~ 1·01	~ 1·01	0·05–0·10

On comparing admissions with major fixed public holidays over the analysis period, we found no strong spike visible on holidays as a group, rather, holidays as a category are associated with slightly reduced admission counts compared with regular days. The largest drop was observed around Christmas with an admission IRR of ~ 0.40–0.50 when compared to regular working days followed by summer vacation with an IRR of ~ 0.60. These reductions were statistically significant with a respective p value of < 0.001 and < 0.01 (Table 2). Weekend admissions and admissions around Easter showed a significant reduction when compared to regular working days. Considering the statistical reduction in the admissions around the vacations, Bonferroni correction applied to multiple comparisons and Cohen’s d was calculated to assess if this reduction was significant. The Coefficient was≈ -0.158 and showed that though the effect (effect size, holiday vs. non-holiday) ≈ -0.158 (small effect).

## Discussion

The results of this 20-year hyperlocal cohort analysis confirm that acute aortic syndromes are non-uniformly distributed throughout the temporal cycle, mirroring trends observed in large-scale multi-centre international registries [4]. The long-term incremental increase in absolute case numbers aligns with the national registries published by the German Society for Thoracic and Cardiovascular Surgery (DGTHG), reflecting aging demographics and improved clinical diagnostic detection [5].

While hindsight is 20/20, foresight is priceless, for everything else, we have analysed the trends in admissions and the patterns that emerged from that analysis.

On separating the dates of admission according to quarters and then months a clear trend emerges. Widespread cardiovascular literature consisting of similar analyses using larger data sets and more robust statistical tests, frequently implicate seasonal meteorological shifts as primary drivers of these cycles. The conventional pathophysiological hypothesis states that exposure to declining ambient temperatures triggers peripheral vasoconstriction and sympathetic activation, leading to acute increases in mean blood pressure and systemic vascular resistance, thereby provoking mechanical failure or dissection of a compromised aortic wall [4, 6].

However, evaluating this environmental hypothesis purely through weather variables reveals several inconsistencies. If declining ambient temperature were the singular dominant trigger, the peak in admission densities, would logically coincide with the absolute coldest months in Bavaria. Historically these have been January and February according to data from the German Weather Service (DWD) [7]. Instead, looking at local datasets, as examined here, demonstrate that the significant epidemiological surge materializes earlier, peaking abruptly in October (IRR = 1.85) and stabilizing through November and December before declining into the deep winter. Furthermore, the stable upward trend in case volume persists despite a documented long-term increase in average regional winter temperatures over the last two decades, suggesting that simple thermal variations are insufficient to fully explain localized disease variation Considering the increase in average winter temperatures over the last 20 years [7] should lead to a drop in the number of acute aortic syndromes, were the temperature the major factor responsible for the variation in the occourance of aortic dissections.

### Considerations of possible underlying sociological factors

Consequently, alternative socio-environmental and institutional hypotheses must be explored. It is not enough to analyse the peaks in the admissions, as they do not follow a normal distribution. The marked reductions in admissions during local public holidays and prolonged school vacations (Christmas, summer, and Easter) point heavily toward regional demographic transitions. The University Hospital Erlangen serves a specific geographic district characterized by low population density and lack of major metropolitan centers as classified by the Globalization and World Cities (GaWC) Research Network 2024 [8]. Given that the median cohort age is 58 years; coinciding with an active working population; these vacation blocks likely experience a significant outbound regional demographic migration or efflux, temporarily shrinking the local population pool at risk for developing acute vascular events.

Conversely, a possible theory for the dramatic surge observed in October may reflect sociological stressors. This period marks the return of the regional workforce from summer recess into high-demand work environments, characterized by compressed year-end deadlines, escalating professional workloads, and impending holiday preparations. Chronic stress and psychosocial acceleration are well-documented contributors to poor blood pressure control and paroxysmal hypertensive crises, which are direct mechanical catalysts for aortic wall disruption.

It is worth remembering that healthcare services are also run by people who are part of the local population. Systemic changes in healthcare availability during holiday seasons cannot be ignored. This may provide another possible explanation as to the peak observed in October. Outpatient medical clinics and primary care providers frequently run at reduced capacity or close entirely during vacation blocks due to personnel leave. This can lead to a temporary interruption of routine healthcare services, resulting in decreased clinical oversight, poor medication adherence, or delayed renewals of critical anti-hypertensive therapies. Subtle adjustments in patient self-monitoring and medication compliance while removed from routine environments may lead to cumulative endothelial and hemodynamic stress that manifests as acute emergencies weeks later in early autumn. Emphasizing the critical importance of medication adherence, particularly uninterrupted anti-hypertensive therapy, and continuous self-monitoring while away from routine medical oversight may contribute to a reduction of modification of risk factors (for e.g. hypertension) in the weeks and months during and after the vacation periods [9–11].

These operational insights have practical implications for modern tertiary care centers. Because acute aortic surgical interventions require massive institutional resources—including specialized surgical staff, perfusion teams, transfusion services, and dedicated intensive care unit (ICU) beds—proactive planning based on cyclical demand timelines rather than static historical averages is highly recommended. Staffing models and elective scheduling could be optimized to maximize institutional resiliency during the documented late-autumn surge.

Over time, the rising numbers in acute aortic syndromes, coupled with rising costs of healthcare and an increased burden on the limited medical personnel means a reduction or redistribution in workload by any and all means should yield positive results in the long run.

The say that good research begets more research. Thus, there are questions the authors find are open after this analysis, namely: would a similar study in a city on another scale of the Globalization and World Cities Research Network classification yield similar results? Does the fall in surgical case load of aortic dissections mean that the patients are not having acute aortic pathologies or does it mean that they are simply occurring elsewhere? What happened in 2017 which led to a high number of cases?

### Limitations and bias

Several limitations must be acknowledged. First, the year 2017 remains a distinct statistical outlier in this series; unlike the general pattern, its peak was driven by an unusual accumulation in Q3 rather than Q4, an occurrence that remains unexplained by local environmental or public data.

Second, because our data source is limited to patients who successfully reached the operating theater, a minor underreporting biases is present regarding out-of-hospital fatalities or patients managed conservatively due to extreme frailty, advanced malperfusion, or advanced age. Post-mortem data indicate that unrecognized AAS can account for up to 7% of sudden out-of-hospital cardiac arrests. [12]. Denying a patient of acute aortic syndrome is, if we believe the data, a decision that ends in a fatality. Surgery is withheld in select cases where risks outweigh benefits, such as in frail elderly patients, those with prior cardiac surgery, organ malperfusion, or refusal of transfusions. These factors often signal underlying pathologies (e.g., severe frailty or comorbidities), which carry a worse prognosis than an acute aortic syndrome, aligning with patient-centred care prioritizing quality of life [13].

Finally, while the identified statistical associations regarding vacation nadirs and autumn surges are robust, the suggested sociological and healthcare system mechanisms remain inferential hypotheses. Confirming these precise links would require granular tracking of personal geographic movements, psychometric stress markers, and primary care compliance data, which exceeds the scope of current clinical data retrieval methods.

## Conclusions

Acute aortic syndromes do not present uniformly throughout the year. An increase in Q4 with a peak in October in our data is consistent with international reports. Vacations and regional holidays show a reduced number of admissions for Acute aortic syndromes. The reasons for this are, as suggested in current literature, not limited to temperature changes but of greater influence could be annual sociological patterns and shifts in the healthcare structure.

## Data Availability

The data used in this study are available upon reasonable request to the corresponding author. The first authors had full access to all of the data in this study and take complete responsibility for the integrity of the data and the accuracy of the data analysis.

## Abbreviations

Q1-4: Quarter 1 to 4 Refers to the quarters of the calendar year (starting in January)
CT-angiography: Computed Tomography Angiography
PAU: Penetrating Aortic Ulcer
DGTHG: Deutsche Gesellschaft für Thorax-, Herz- und Gefäßchirurgie
DWD: Deutscher Wetterdienst (German Weather Service)
AAS: Acute Aortic Syndromes
